# A Case Report of an Unusual Presentation of a Pedunculated Gastrointestinal Stromal Tumor: Acute Upper Gastrointestinal Bleeding in a 57-Year-Old Female

**DOI:** 10.7759/cureus.47562

**Published:** 2023-10-24

**Authors:** Atheer Almutairi, Alanoud Almuhana, Rahaf Alanazi, Ali AlZughbi, Abduljaleel Al Alwan

**Affiliations:** 1 College of Medicine, King Saud Bin Abdulaziz University for Health Sciences, Riyadh, SAU; 2 Medicine, King Saud Bin Abdulaziz University for Health Sciences, Riyadh, SAU; 3 Medicine and Surgery, King Saud Bin Abdulaziz University for Health Sciences, Riyadh, SAU; 4 Pathology and Laboratory Medicine, King Fahad Medical City, Riyadh, SAU; 5 Hepatology, King Abdulaziz Medical City, Riyadh, SAU

**Keywords:** interstitial cells of cajal, leiomyosarcomas, git endoscopy, gastrointestinal stromal tumor (gist), upper gi bleeding

## Abstract

Gastrointestinal stromal tumors (GISTs) are rare tumors accounting for 0.1-3% of gastrointestinal (GI) neoplasms. ‏In the past, GIST was classified as leiomyomas, leiomyosarcomas, and leiomyoblastomas. However, now it is evident that GIST is a separate tumor entity, and it is the most frequent sarcoma of the GI tract. We report a case of a 57-year-old female with a five-day history of black tarry stools, two episodes of vomiting of dark-colored blood, dizziness, abdominal pain, night sweats, and palpitation, provoked by a change of position. After a computerized tomography (CT) of the abdomen and pelvis, a GIST was suspected, which was confirmed with histopathology. Acute upper GI bleeding is a rare presentation of GIST. Clear guidelines should be developed for GIST. An early diagnosis is crucial for a better prognosis.

## Introduction

Gastrointestinal stromal tumors (GIST) are rare tumors. They are considered a subtype of the mesenchymal tumors of the gastrointestinal (GI) tract accounting for 0.1-3% of GI neoplasms. GISTs were believed to arise from smooth muscles. However, evidence indicates that these tumors originate from stem cells that differentiate into the interstitial cells of Cajal, or directly from the interstitial cells of Cajal [1]. The interstitial cells of Cajal are considered a part of the myenteric plexus in the GI tract and are responsible for regulating peristalsis [2]. The most frequent locations of GIST are the stomach 50-70%, the small intestine 20-30%, the colon and rectum 5-15%, and less than 5% in the esophagus. Most patients present in the sixth decade, with abdominal pain, nausea, vomiting, early satiety, and dyspepsia. However, in some patients, GISTs can be a source of intraperitoneal hemorrhage (61%) or can cause bleeding in the GI tract lumen, resulting in melena, hematemesis, or anemia. GIST can be classified histologically in spindle cell type (70%), epithelioid cell type (20%), and mixed type (10%). Surgery is the definitive therapy for GIST. In this article, we report a 57-year-old female who was diagnosed with mixed GIST [3].

## Case presentation

History

We report a case of a 57-year-old female who presented to a private hospital with a five-day history of acute black tarry stools, two episodes of vomiting of dark-colored blood, dizziness, abdominal pain, night sweats, and palpitation, provoked by a change of position as laying down improved these symptoms. According to the patient, the symptoms were relieved by lying down and drinking water. During the hospital admission, she also reported developing stabbing abdominal pain that was mildly relieved with water intake and associated with nausea and multiple episodes of extensive black bloody vomiting. The patient reported a history of weight loss (9kg) since the onset of symptoms. She had a past medical history of hyperlipidemia. Her past surgical history was not clear. She denied any recent travel, contact with sick patients, smoking, and the use of alcohol or drugs. The patient developed uncontrollable smelly bloody diarrhea. The patient denied any history of heartburn, pain radiating to the back, or pain aggregated or relieved by meals.

Examination

The patient was vitally and clinically stable. The patient was afebrile, pallor was not noted on the skin or the eyes. On examination, the patient had mild epigastric abdominal pain and tenderness. The patient had no signs of jaundice, encephalopathy, edema, or caput medusa, which excluded liver disease or portal hypertension. There was no abdominal destination, or rebound tenderness. The bowel sounds were normal. The cardiac examination was insignificant with a normal S1 and S2.

Investigations

The patient was suspected to have a GIST. An endoscopy was used to provide an accurate evaluation, which indicated an isolated fundus varix and non-active esophageal varix.

**Figure 1:**
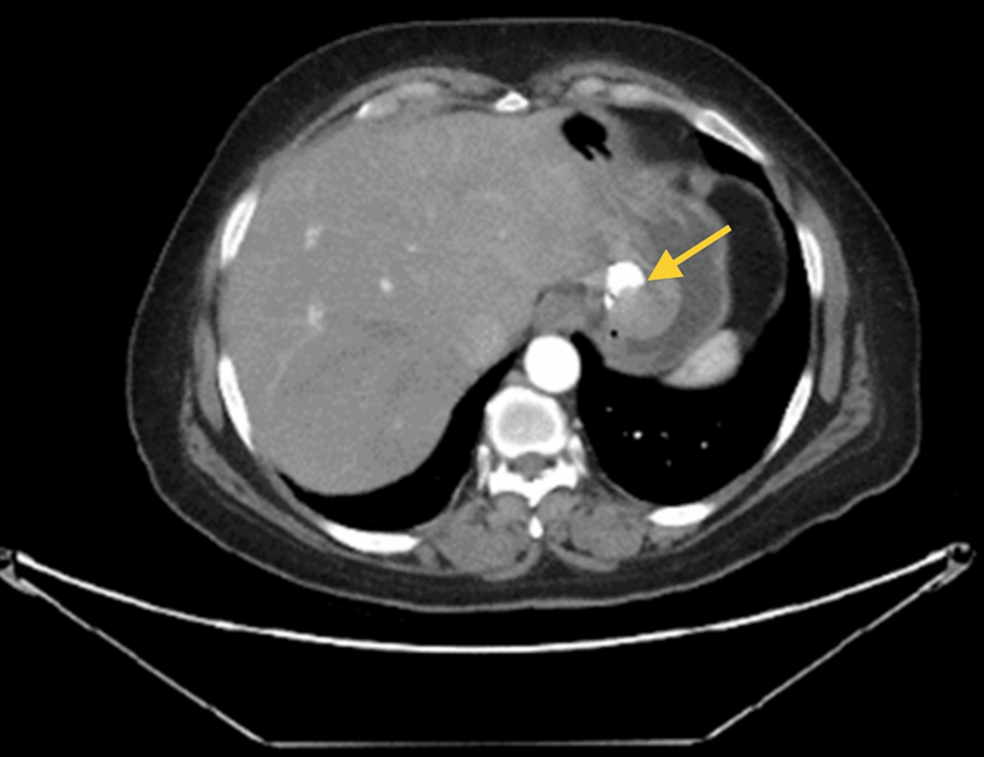
Well-defined rounded submucosal pedunculated structure arising from the gastric fundus measuring 3.4 x 3.2 x 3 cm (arrow).

**Figure 2:**
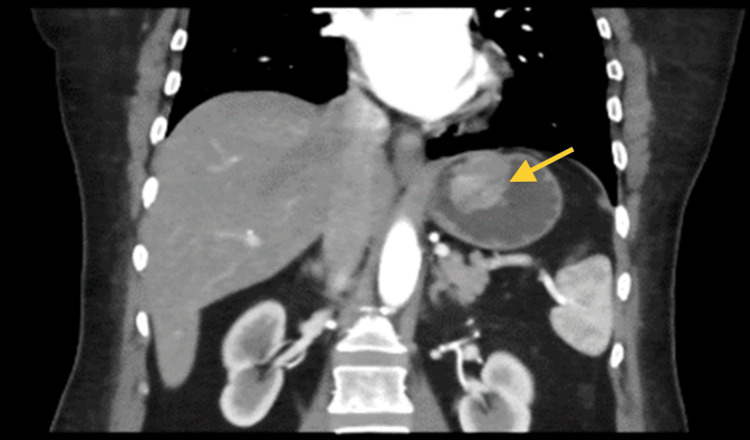
In the coronal view, a submucosal pedunculated structure originating from the gastric region was observed (arrow), indicating the presence of a GIST. GIST: Gastrointestinal stromal tumor

A computerized tomography (CT) of the abdomen and pelvis was done, and the results showed a submucosal/endophytic gastric fundus heterogeneously hyperdense structure (Figures 1-2). A CT scan shows a well-defined rounded submucosal pedunculated structure arising from the gastric fundus measuring 3.4 x 3.2 x 3 cm.

**Figure 3:**
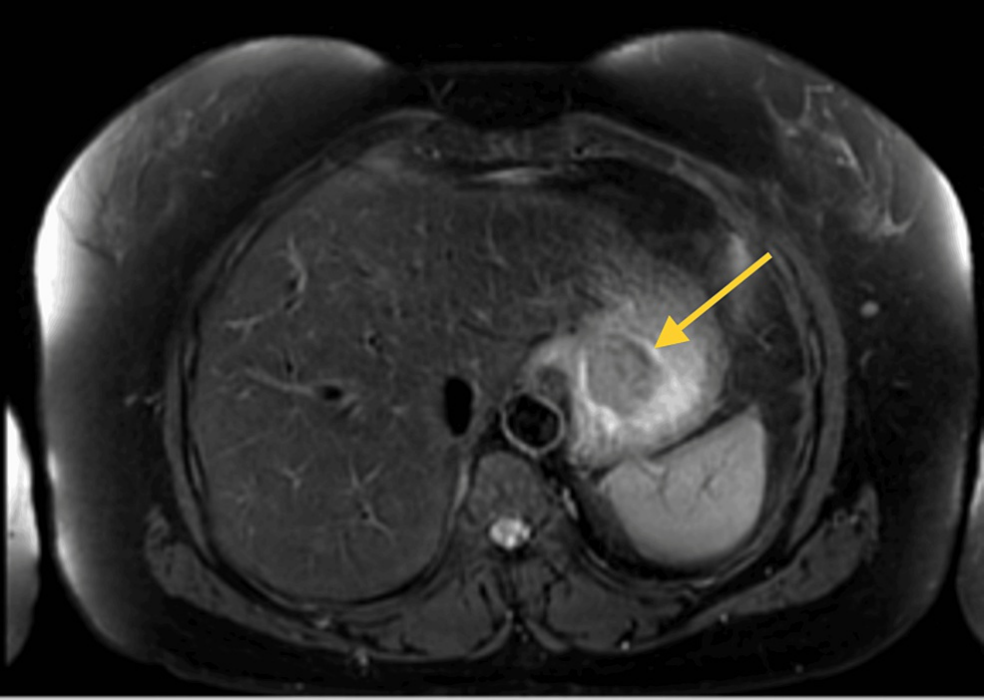
An abdominal MRI revealed the presence of a gastric fundus mass, indicative of a GIST (arrow). GIST: Gastrointestinal stromal tumor

The diagnosis was confirmed through magnetic resonance imaging (MRI) of the abdomen, indicating GIST (Figure 3).

**Figure 4:**
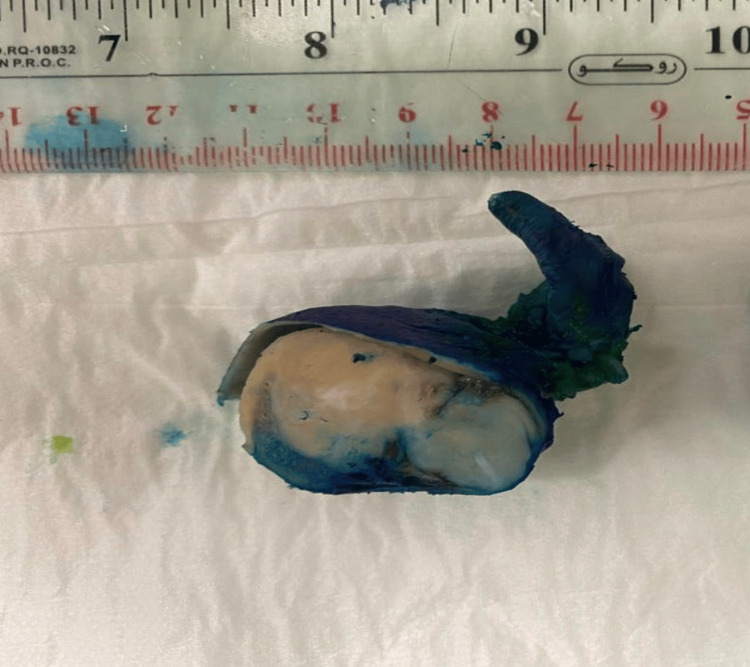
Specimen We received this specimen in formalin containing a 6.5 x 5.5 x 3.5 cm fundic mass.

We received this specimen (Figure 4) in formalin containing a 6.5 x 5.5 x 3.5 cm fundic mass, the tumor is solitary, well-circumscribed, and fleshy with no necrosis identified. The gastric mucosa overlying the tumor is normal and no ulcer is seen.

**Figure 5:**
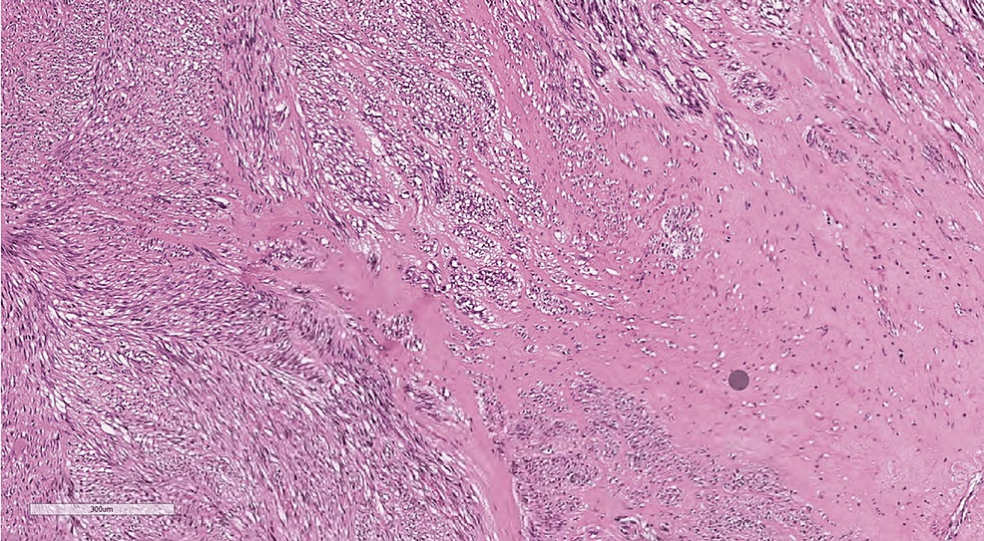
Histology Histology reveals the tumor was composed of cells with spindle (elongated nuclei and eosinophilic cytoplasm arranged in fascicles) and epithelioid (mildly pleomorphic epithelioid cells with abundant eosinophilic cytoplasm) morphology.

**Figure 6:**
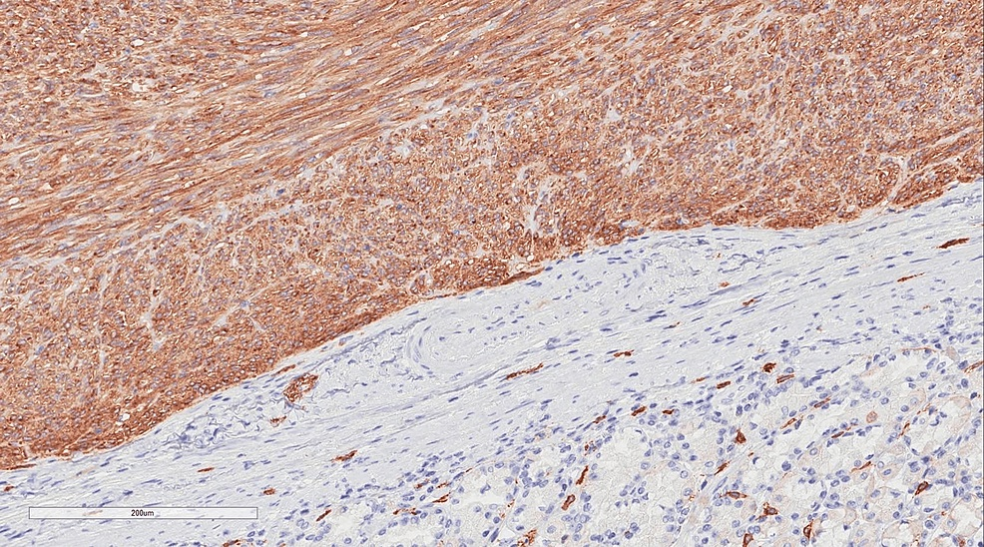
Immunobiological staining revealed a positive expression of CD117 in the neoplastic cells.

**Figure 7:**
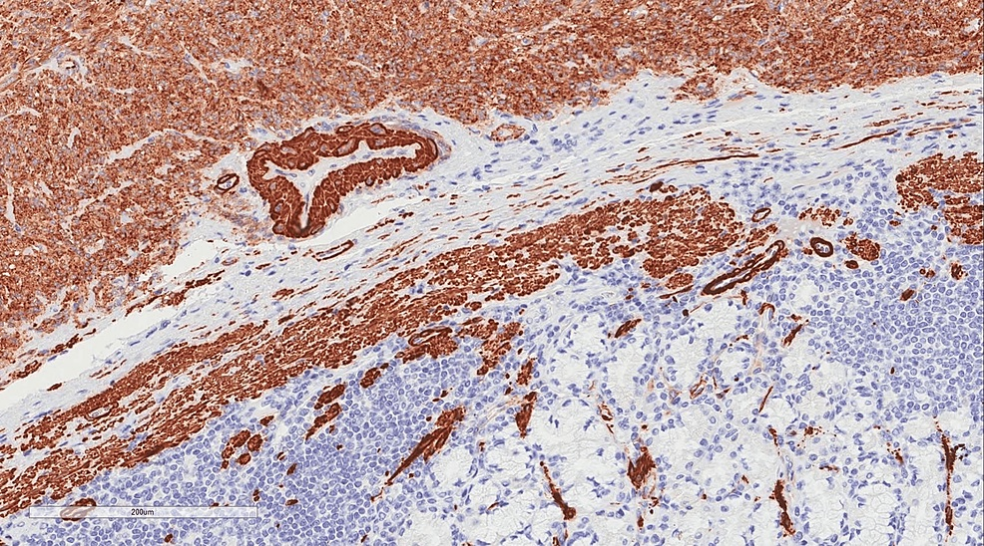
Immunobiological staining revealed a positive expression of Caldesmin in the neoplastic cells.

**Figure 8:**
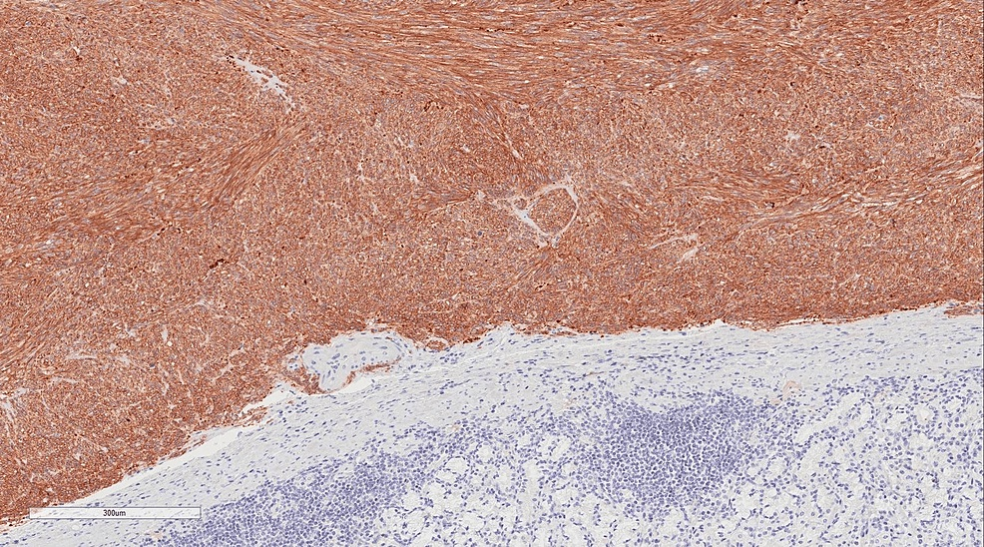
Immunobiological staining revealed a positive expression of DOG-1 in the neoplastic cells.

Immunobiological staining was used to differentiate between the types of GIST. In our patient, the caldesmon stain was positive, which is only observed in 10% of all the cases (Figures 5, 6, 7, 8).

The neoplasm is positive for DOG1, Caldesmon, and CD117. It's negative for Ki-67.

Surgical intervention, outcome, and follow-up

Considering the radiological and histopathological findings, the patient underwent a laparoscopic partial fundus resection as it was appropriate for this case. The tumor was resected to a negative margin of 1 cm distal to the mass. The patient tolerated the procedure well and was transferred to the post-anesthesia care unit (PACU) in stable condition. The patient was discharged in a stable condition and instructed to come to the ER in case of severe pain, fever, or vomiting. An outpatient appointment was scheduled after six months for clinical and radiological follow-up.

## Discussion

GISTs, previously classified as leiomyosarcomas due to their resemblance to smooth muscles, based on immunohistochemistry, have been recognized as stromal tumors associated with the expression of neural crest cell antigens in 1984. The cellular origin of GITS is thought to be from mesenchymal stem cells, programmed for differentiation into the interstitial cell of Cajal. These cells initiate coordinated GI motility and are the pacemakers of the GI system [4]. Population-based studies from European countries, in addition to SEER (surveillance, epidemiology, and results) data from the United States, indicate an annual incidence rate of 6.5% to 14.5% and an age-adjusted incidence rate of 0.68 to 0.8 per 10,000. Unfortunately, the global incidence of GISTs is not known, given the relative homogeneity of the previous population-based studies. GISTs most frequently are diagnosed later in life; however, they can occur at any age. Shenoy and Singh reported a neonatal boy diagnosed with GIST in the terminal ileum after presenting with intestinal obstruction and vomiting one day after his birth [5]. To current knowledge, there is one report of solitary gastric pseudo-variceal rupture caused by pedunculated GISTs of a 64-year-old male [6]. He was admitted to the emergency department due to episodes of hematemesis and melena, and presented with normal blood pressure (120/90 mmHg) and tachycardia (102 beats/min). His medical history is negative for peptic ulcers and liver diseases which is similar to the the presentation of our patient. An upper endoscopy was performed, which showed Bluish-bloated gastric mucosal folds of the greater curvature with a string-of-beads aspect and a red spot. These findings mimicked the post-ruptured status of solitary gastric varices (GVs). However, the endoscopy in our patient showed no active (non-bleeding) isolated fundal varies. CT scan revealed an irregular pedunculated mass measuring approximately 60 mm in diameter on the serosal side of the stomach, the enhanced mucosal side of the tumor suspected of being vascular-enriched gastric submucosa. In our case, the CT scan showed a well-defined rounded submucosal pedunculated structure arising from the gastric fundus measuring 3.4 x 3.2 x 3 cm.

Diagnosis

The location of GIST impacts the diagnostic workup involved. In all patients, regardless of the presenting symptoms, a history and a physical examination should serve as the starting point for the diagnostic work-up. Most frequently, patients with these tumors present with anemia and other signs and symptoms of chronic GI bleeding. Patients rarely present with acute GI bleeding manifesting as melena or hematochezia [7]. We believe that such an unusual presentation is associated with the development of isolated fundal varices in association with GIST as in the case of our patient. This case is important because it illustrates the significance of keeping mixed GIST as one of the differentials for patients presenting with acute upper GI bleeding especially since previous studies had demonstrated the consequences of delayed surgery in these patients. In addition to GI bleeding, GISTs may also present with signs and symptoms of a mass effect caused by the tumor, such as abdominal pain or discomfort, early satiety, abdominal distension, or a palpable mass. In an additional 15% to 30% of cases, GISTs are found incidentally during surgery, imaging, or autopsy [8]. Gastroscopy, endoscopic ultrasound, and abdominal and pelvic imaging support the diagnosis. The final diagnosis is based on the pathological and immunohistochemical examination. Immunohistochemical staining for the CD 117 tyrosine kinase receptor, which reveals the presence of interstitial cells of Cajal, is used to confirm the diagnosis of GIST. The expression of CD 117 distinguishes GISTs from stomach schwannomas and genuine leiomyomas, which consistently test negative for CD 117. In about two-thirds of GISTs, CD 34 is expressed. A crucial confirming sign for the diagnosis of this malignancy is CD117. These tumors may display a mixed subtype, an epithelioid pattern, or a spindle cell pattern histologically [9]. In our case, the histopathology revealed a spindle cell pattern. Tumor size and mitotic rate are the most important tumor factors for local recurrence and metastasis. The risk of recurrence and metastasis increases with a tumor size of more than 5 cm and mitosis > 5 per 50 HPF. The histopathology report of our patient was low-risk benign GIST. A case report of Alkhaldi described a seven-year-old female presenting with a four-month duration of pallor accompanied by a low hemoglobin of 5 and severe hypochromic anemia. An upper endoscopy and barium swallow were negative. The patient was managed with blood transfusion and iron supplements. After a recurrent episode of the same complaint, a CT was done that showed a 3cm upper posterior fundal mass and GIST was confirmed by immunohistochemistry. Recently, many GIST cases have been reported in pediatrics, and it is important to have clear guidelines regarding the diagnosis and management of pediatric-onset GIST. No current guideline exists. It is important to consider GIST as a differential diagnosis for pediatric patients presenting with signs and symptoms of chronic anemia.

Treatment

The treatment approach for GI stromal tumors depends largely on the size and stage of the tumor. When dealing with a localized tumor that can be surgically removed and is larger than 2 cm, the primary treatment method is surgical resection [10]. However, for patients with locally advanced disease where complete surgical removal is not feasible without causing functional impairment, the use of preoperative imatinib therapy can help reduce the tumor size prior to surgery. In cases where the disease is considered high-risk, adjuvant therapy is recommended, typically involving three years of tyrosine kinase inhibitors, preferably imatinib [11]. When the tumor is unresectable or has spread to other parts of the body, treatment with tyrosine kinase inhibitors is recommended [12]. In the case of our patient with a tumor measuring 3.5 x 3.6 x 2.4 cm, the treatment plan involved complete tumor removal with clear margins, and postoperative therapy was deemed unnecessary based on the biopsy results. However, it is important to note a previous case where delayed therapy resulted in significant consequences. In that case, a 51-year-old female with a GIST measuring 102 x 56.3 x 46.6 mm experienced a delay of five months in undergoing surgery and receiving medical treatment due to COVID-19 regulations. As a result, the patient suffered from upper abdominal pain and black tarry stools, eventually leading to severe bleeding and symptoms such as fatigue and syncope. Her hemoglobin level dropped significantly from 11 mg/dL to 5.5 mg/dL over a period of four months [13].

Prognosis

The outlook for individuals diagnosed with GI stromal tumors depends on factors such as where the tumor is located, the rate of cell division (mitotic count), and the size of the tumor. Additional factors that influence the prognosis include whether the tumor is completely removed with no residual cancer cells at the surgical margins and whether there was a rupture of the tumor during the surgical removal. Common complications associated with GI stromal tumors include GI bleeding and the physical pressure exerted by the tumor. Chronic GI bleeding caused by GIST is the most frequent complication and can lead to anemia. Furthermore, these tumors can also result in intestinal blockage, bleeding within the abdominal cavity, and rupture followed by inflammation of the abdominal lining (peritonitis) [13].

## Conclusions

Mixed GISTs are rare, especially when presenting as acute GI bleeding. GIST presenting acutely can be misdiagnosed initially as isolated gastric fundal varices. Radiological imaging plays a major role in diagnosing the condition. Different treatment options are considered, such as medical and surgical therapy. Although the mechanism behind the development of GVs is known, the association between GVs and GIST needs further studies in terms of prevalence, management, and complications.
